# Human fusariosis: An emerging infection that is difficult to treat

**DOI:** 10.1590/0037-8682-0013-2020

**Published:** 2020-06-01

**Authors:** Bruna Gerardon Batista, Magda Antunes de Chaves, Paula Reginatto, Otávio Jaconi Saraiva, Alexandre Meneghello Fuentefria

**Affiliations:** 1 Universidade Federal do Rio Grande do Sul, Programa de Pós-Graduação Stricto Sensu em Ciências Farmacêuticas, Porto Alegre, RS, Brasil.; 2 Universidade Federal do Rio Grande do Sul, Programa de Pós-Graduação em Microbiologia Agrícola e do Ambiente, Porto Alegre, RS, Brasil.; 3 Universidade Federal do Rio Grande do Sul, Faculdade de Farmácia, Departamento de Análises, Porto Alegre, RS, Brasil.

**Keywords:** *Fusarium* spp, Emerging fungal infection, Human fusariosis

## Abstract

*Fusarium* spp. has been associated with a broad spectrum of emerging infections collectively termed fusariosis. This review includes articles published between 2005 and 2018 that describe the characteristics, clinical management, incidence, and emergence of these fungal infections. *Fusarium solani* and *F. oxysporum* are globally distributed and represent the most common complexes. Few therapeutic options exist due to intrinsic resistance, especially for the treatment of invasive fusariosis. Therefore, the use of drug combinations could be an important alternative for systemic antifungal resistance. Increase in the number of case reports on invasive fusariosis between 2005 and 2018 is evidence of the emergence of this fungal infection.

## INTRODUCTION

Fusariosis is an infection that affects plants, animals, and humans, and is caused by various fungi of the genus *Fusarium* ^1^ ^,^ ^2^. *Fusarium* spp. is responsible for significant economic losses in the agricultural field worldwide^3^due to difficulties in management of diseases caused by this species^4^. Similarly, in the medical field, different *Fusarium* species have been related to local or invasive infections in both immunodepressed and immunocompetent individuals^5^ ^,^ ^6^ ^,^ ^7^.

However, infections are difficult to treat because of the lack of consensus regarding treatment protocols for fusariosis in humans caused by multi-drug resistant isolates^2^ ^,^ ^8^ ^,^ ^9^. In addition, it is possible that environmental isolates from *Fusarium* spp. acquire resistance due to previous exposure to fungicides that were used in the agricultural fields^10^ ^,^ ^11^, and these isolates may disseminate and consequently infect humans^12^ ^,^ ^13^. Perhaps this process of infection may be avoided by implementing public control policies regarding the sale and use of fungicides.

As such, the aim of this study is to review the literature to demonstrate the characteristics, clinical management, incidence, and emergence of fungal infections caused by *Fusarium* species. The lack of attention on these cases by public health institutions and the insufficient research on the development of novel antifungal agents as therapeutic options emphasize the need to address the main factors involved in fusariosis, such as clinical forms, treatment, and lack of epidemiological control. To address this problem, articles published between 2005 and 2018 were analyzed, and 23 publications were obtained that included important conclusions regarding this proposition (Table 1).

**TABLE 1:** Timeline of fusariosis: publication basis for the review study and conclusions of the last 15 years.

Authors	Year	Journal	Main conclusions
Nucci and Anaissie	2007	Clinical Microbiology Reviews	Infections by the *Fusarium* species are superficial in healthy patients, and these patients
			tend to respond well to therapy. Disseminated fusariosis affects the
			immunocompromised host and is often fatal.
Katiyar and Edlind	2009	Antimicrobials Agents and	Genetic mutations in Fks sequences result in decreased sensitivity of the *Fusarium* sp.,
		Chemotherapy	rendering it difficult to treat human fusariosis.
Romani	2011	Nature Reviews Immunology	When the infective structures of *Fusarium* spp. reach the mucous membranes, the
			innate cellular immune response of the host is activated, which includes dendritic cells,
			macrophages, monocytes, neutrophils, and soluble mediators of the complement system.
Guarro et al.	2013	European Journal of Clinical	Fusariosis is related to high mortality. Recovery from neutropenia remains the most important determinant of outcomes in such patients.
		Microbiology & Infectious Diseases	
Nucci et al.	2014	Clinical Microbiology and	Significant improvement in the results of invasive fusariosis in the last decade with
		Infection	changes in therapeutic practices, involving a decrease in the use of amphotericin B and
			increase in the use of voriconazole and combination therapy.
Spolti et al.	2014	Plant Disease	Epidemic of fusariosis in plants can be harmful to humans and animal health, since the
			ingestion of cereals contaminated with mycotoxins can cause serious food poisoning.
van Diepeningen et al.	2014	Current Clinical Microbiology Reports	The use of molecular techniques is recommended to identify *Fusarium* species that cause infections.
Varon et al.	2014	The Journal of Infection	Skin lesions may be considered entry points for *Fusarium* spp. Infections, especially in
			individuals that exhibit risk factors, such as high-risk hematological patients.
Price et al.	2015	Pest Management Science	*Fusarium* sp. exhibits mechanisms that contribute to the acquisition of resistance to
			even the most diverse antifungal agents. These mechanisms include changes in the
			amino acid sequence, overexpression of the *CYP51* gene, and overexpression of
			genes that encode efflux pumps.
van Diepeningen et al.	2015a	Current Fungal Infection	Different *Fusarium* species have been associated with local or invasive infections in both
		Report	immunosuppressed and immunocompetent individuals.
van Diepeningen et al.	2015b	Mycoses	Members of the *F. solani* complex are the most common and virulent, followed by *F.*
			*oxysporum*, *F. fujikuroi,* and *F. moniliforme*.
Al-Hatmi et al.	2016a	Emerging Microbes & Infections	Treatment given for *Fusarium* infections varies according to the site of infection.
Al-Hatmi et al.	2016b	The Journal of Antimicrobial	*In vitro* combined use of natamycin and voriconazole was found to be synergistic
		Chemotherapy	against most *Fusarium* strains, thereby significantly reducing the concentrations
			required to inhibit fungal growth.
Dalhoff	2016	Journal of Global Antimicrobial	Fusariosis is difficult to treat and the use of antimycotics in agriculture and horticulture
		Resistance	facilitates the acquisition of antifungal resistance.
Espinel-Ingroff et al.	2016	Antimicrobial Agents and	A cutoff point for minimum inhibitory concentration values for various *Fusarium* species
		Chemotherapy	was proposed based on laboratory results.
Ribas et al.	2016	Brazilian Journal of	Environmental isolates of *Fusarium* spp. could acquire resistance due to previous
		Microbiology	exposure to fungicides that are used agriculturally in the field.
Al-Hatmi et al.	2017	Journal of Fungi	No standardization is established regarding MIC cut points for *Fusarium*, which renders
			it difficult to classify the susceptibility profile of isolates.
Batista et al.	2017	Chemistry Select	New chemical molecules exhibited low MICs (high potency) against *Fusarium* spp. and
			reduced toxicity with promising applicability in the biological and industrial fields.
Fuentefria et al.	2017	Letters in Applied	Combination therapy have been an important alternative for combating *Fusarium* species
		Microbiology	.
Kolar et al.	2017	Investigative Ophthalmology &	Dectin-1 and TLR2 play an important role in the regulation of *F. solani-*induced AMP
		Visual Science	expression in corneal epithelial cells, facilitating the eradication of fungal pathogens.
Al-Hatmi et al.	2018	International Journal of	New identification tools for *Fusarium* spp. to aid in selecting the most appropriate treatment
		Antimicrobial Agents	.
Bashir et al.	2018	Environmental Science and	Evaluated various concentrations of fungicides used to combat fusariosis in plant. The
		Pollution Research	use of carbendazim significantly reduced the incidence of disease by 49.7% after 40
			days of application.
Homa et al.	2018	Frontiers in Microbiology	*F. falciforme* was the most prevalent species of FSSC in South India. Susceptibility and
			virulence of clinical and environmental isolates were similar.

## EMERGENCE OF PATHOGENIC FUSARIUM SPECIES

*Fusarium* species exhibit global distribution, and it is believed that approximately ten complexes are related to human pathogens, including *F. solani*, *F. oxysporum*, *F. fujikuroi*, *F. incarnatum-equiseti*, *F. clamydosporum*, *F. dimerum*, *F. sambucinum*, *F. concolor,* and *F. lateritium* ^5^ ^,^ ^14^. Among these complexes, members of the *F. solani* complex are the most common and virulent (comprising approximately 40-60% of infections), followed by *F. oxysporum* (~20%), *F. fujikuroi* and *F. moniliforme* (~10%)^5^ ^,^ ^14^ ^,^ ^15^. 

Despite global distribution, endemic regions are tropical and subtropical in nature^7^ ^,^ ^16^. Although fusariosis is associated with specific climatic conditions, environmental and clinical isolates have been reported to cause infections outside previously established borders^14^. This fungus has efficient mechanisms of dispersion, and its conidia reach considerable distances^9^. Moreover, genetic similarities between clinical isolates and environmental isolates of the same species may be related to infections in patients by *Fusarium* spp. in the environment^16^.

## CLINICAL ASPECTS OF FUSARIOSIS

*Fusarium* species cause a wide spectrum of infections in humans, ranging from superficial and locally invasive to disseminated, with the most prevalent infections being onychomycosis, skin infections, and keratitis^15^.

Invasive infections can be widespread involving the skin, brain, bloodstream, lungs, eyes, and bones^14^ ^,^ ^17^ ^,^ ^18^. Patients with severe and prolonged neutropenia, especially those with hematological malignancies, are most susceptible to prevalent infections^9^ ^,^ ^18^.

In their epidemiological study, Garnica and Nucci described the worldwide incidence of *Fusarium* spp. as the main non-dermatophyte filamentous fungus that causes onychomycosis^19^. These infections are presented as subungual distal and total dystrophic infections that are often associated with paronychia and characterized by purulent periungual inflammation. The most commonly involved complexes are *F. oxysporum* and *F. solani* ^20^. Treatment is difficult and prolonged, usually lasting more than 4 or 6 months, even with the use of topical and systemic antifungal agents^21^.

Keratitis is one of the most common infections caused by *Fusarium* spp. and primarily develops from trauma to the eye, contact lens wear, and use of corticosteroids^9^ ^,^ ^19^ ^,^ ^22^. Trauma is the key predisposing factor and occurs in 40-60 % of patients^19^. 

Skin infections are the result of dissemination of the fungus primarily in immunocompromised patients^14^. The most common pattern of disseminated disease is the combination of multiple painful erythematous papules or nodules, commonly with central necrosis. Such occurrences spread throughout the body and continuously release fungal cells, thereby resulting in a positive blood culture, and often pulmonary involvement, with or without involvement at other sites^9^ ^,^ ^17^.

The airways represent the main gateway to infection, followed by the skin at the site of the tissue or onychomycosis, contact lens wear, and possibly mucous membranes^9^ ^,^ ^18^ ^,^ ^22^. Prior to initiating immunosuppressive therapy- given the severity associated with disseminated fusariosis- signs of the presence of skin or nail infections should be carefully investigated, as they comprise the focus of fungal dissemination and are often neglected upon initial physical examinations^5^ ^,^ ^9^ ^,^ ^22^. 

## IMMUNE RESPONSE AGAINST FUSARIOSIS

The immune system impedes the establishment of invasive infections by various species of fungi as high mortality is seen in immunosuppressed individuals^23^. However, in terms of the emerging pathogenic species of the genus *Fusarium*, the lymphocyte response via Th2 may facilitate the invasiveness of this disease and explain the self-limiting difficulty related to its complex mycosis^24^. 

When the infective structures of *Fusarium* spp. reach the mucous membranes, the innate cellular immune response of the host is activated, which includes dendritic cells, macrophages, monocytes, neutrophils, and soluble mediators of the complement system^25^. These responses are initiated by pattern recognition receptors (PRRs), which recognize a series of common and constant molecular patterns that are present in nearly all microorganisms, denominated as pathogen-associated molecular patterns (PAMPs). The activation of PRRs plays a dual role: it initiates processes of the innate immune system, such as phagocytosis, and establishes a link between innate and adaptive immunity via MHC type I and type II expressions^25^_._

The most important PAMPs in filamentous fungi are mannan, β-glucan, and chitin. The primary soluble PRR is pentraxin-3, whereas cellular PRRs are lectins, Toll-like-receptors, and NOD receptors. *Fusarium* species are recognized by type 2 Toll-like-receptors, which are generated in response to the production of anti-inflammatory cytokines (IL4 and IL10), and thus promote an adaptive immune system response that is mediated by Th2 lymphocytes^26^ ^,^ ^27^. Thus, invasive *Fusarium* infections stimulate a Th2-type lymphocyte response, in which anti-inflammatory cytokines are produced, thereby leading to an inadequate response by the host to the infection and high morbidity and mortality^28^.

Despite their minor importance, various humoral factors also participate in the innate response, as the complement is activated by their associated classical and alternating pathways^25^. However, the predisposing factors of invasive mycoses relate to the dysfunction of the immune system of phagocytosis, rather than defects in humoral immunity. More knowledge on humoral immunity activity in response to fungal infections is required, although some studies have attempted to demonstrate a specific marker of invasive diseases caused by *Fusarium* spp.^29^.

## ANTIFUNGAL RESISTANCE AND THERAPEUTIC OPTIONS

*Fusarium* spp. exhibit intrinsic resistance to echinocandins^2^. Moreover, some isolates exhibit resistance to azoles that are associated with a third analogue of the *CYP51* gene^30^. On the other hand, the intrinsic resistance of echinocandins is linked to the Y639 region of the *FKS1* gene, which is responsible for encoding the catalytic subunit of β-1-3 glucan synthase^31^. These fungi also exhibit mechanisms that contribute to acquiring resistance to most diverse antifungal agents, such as changes in amino acid sequences, overexpression of the *CYP51* gene, and overexpression of genes that encode efflux pumps^32^. 

Minimal inhibitory concentrations and minimum effective concentrations have not been established for *Fusarium* species^20^. To present this missing knowledge, Espinel-Ingroff^8^ defined the epidemiological breakpoints for amphotericin B, posaconazole, and itraconazole in relation to the main *Fusarium* species that cause fusariosis. In this scenario, a few options exist to combat this infection, and the frequently used antifungal agents include natamycin, amphotericin B, voriconazole, and posaconazole^5^. Therefore, depending on the clinical case, amphotericin B and voriconazole are the drugs of choice^9^ ^,^ ^33^. *In vitro* and *in vivo* tests also reveal natamycin and voriconazole as drugs of choice to treat keratitis induced by *Fusarium* spp.^2^

In the case of resistance, the use of combinations of drugs may be an important alternative to combat various *Fusarium* species, increase the efficacy and spectrum of action of antifungal agents, and lower drug dosage and thus reduce toxic side effects^34^ ^,^ ^35^. Moreover, *in vitro* drug combinations have demonstrated the ability to control fungal biofilms in other fungal species^36^, and studies focused on *Fusarium* sp. remain scarce. Combinations of antifungal and non-antifungal agents have also been tested *in vitro* and the results are promising, especially in fusariosis, as a strong association with the inflammatory response has been found^37^ ^,^ ^38^. Despite promising results in an *in vitro* context, the use of combinations requires clinical studies to verify its effectiveness *in vivo*. A few reports have been conducted on treating patients with fusariosis using more than one drug. Tortorano et al. (2014) have reported an association between the use of lipid-based amphotericin B and voriconazole, as well as the use of up to three antifungals in the same patient^33^.

Factors that contribute to the severity of fusariosis include increased incidence of multidrug resistance to *Fusarium* spp.^39^ and the lack of research relating to the development of new therapeutic options for treatment. In general, these infections progress with a severe prognosis, especially in terms of ophthalmology, in which cases of fungal keratitis led to negative outcomes, such as loss of vision, in affected individuals. Currently, isavuconazole, characterized as a second generation triazole antifungal, is being studied as an alternative for its potential treatment of fungal diseases in patients with hematological diseases^40^.

## FUNGICIDES AND RESISTANCE IN PHYTOPATHOGENIC FUSARIUM SPECIES

Fungicides are specific substances that are used in the agricultural field to combat and prevent fungal diseases. Waste from the use of these substances is considered a pollutant with potential risk to the human body, as well as more commonly to the environment^41^. Demethylation inhibitors are abundantly used in the agricultural field. Moreover, demethylation inhibitors change the fungal population after multiple applications, thus requiring the application of new fungicides. A substitute used is triazole, and its time of permanence in the soil depends on the concentration used and generally ranges from 67 days to more than 1688 days, with a trend of accumulation based on the frequency of use^42^.

A risk factor that may be associated with fungicides in the environment is the development of microbial resistance^4^ similar to that associated with the overuse of antifungals in humans^30^. Azoles are the most commonly used of all groups for both pest control and treatment of human infections. Therefore, the potential development of resistance to this specific class is of increasing concern^4^. Some benefits of the azole class include low cost and high efficacy, thereby rendering it the first-choice antifungal for use as a fungicide agent in crops since the 1970s^32^. 

Proper fungicide management in agricultural fields is a current demand in terms of the economics related to agricultural practices, as well as in terms of negative environmental impact^43^.

## INCIDENCE OF HUMAN FUSARIOSIS

Cutaneous lesions have been observed due to the spread of fungi in patients with hematological diseases. In Brazil, from 2007 to 2009, invasive fusariosis was proved to be the most frequent or probable invasive fungal disease, with 23 episodes among 937 patients with hematologic diseases^19^. Based on the information discussed thus far, a bibliographical search was conducted on the PubMed and Science Direct platforms using the term "fusariosis in human," including case reports published between 2005 and 2018. In this review, we included data from articles published only in 2005 and 2018, comprising 14 publications, with the aim to observe possible changes in both the etiology of infections and treatment (Table 2). The factors for inclusion of the case reports involve the presence of relevant information on etiological agents, predisposing factors, and treatments. The exclusion factor was defined as the lack of any required information, as previously cited.

**TABLE 2:** The symptoms of patients, treatments, etiological agents, and risk factors for patients described in articles published in 2005 and 2018.

Author	Year	Symptoms of Patients	Treatment	Etiological Agent	Risk Factors
Hayashida et al.^45^	2018	Erythematous nodules	Amphotericin B and	*F. solani*	Acute myeloid leukemia
			voriconazole		
Simon et al.^46^	2018	Pain and decreased vision	Amphotericin B and voriconazole	*F. dimerum*	Acute myeloid leukemia
Boral at al.^47^	2018	Blurred vision	Voriconazole	*F. solani*	Ocular trauma
Combalia et al.^48^	2018	Lesions on the	Complete excision of	*F. solani*	Diabetes mellitus
		eyebrow	the lesions		Kidney transplant
					Immunosuppress treatment
Okada et al.^49^	2018	Lesions forming an	Liposomal	*F. solani*	Neutropenia
		ulcer	amphotericin B		Varicella zoster virus
Puapatanakul et al.^50^	2018	Peritonitis and septicemia	Amphotericin B	*Fusarium* spp.	Diabetes mellitus Hypertension
					End-stage kidney disease
Borges et al.^51^	2018	Lesion	Amphotericin B and	*F. solani*	Acute myeloid leukemia
			itraconazole		Neutropenia
Arnoni et al.^52^	2018	Nodules on the chest	Amphotericin B and voriconazole	*F. oxysporum*	Acute lymphocytic leukemia
Kumari et al.^53^	2018	Lesions with pus discharge	Itraconazole	*F. solani*	HIV positive
Yoshida et al.^54^	2018	Blurred vision	Amphotericin B and voriconazole	*F. solani*	Acute myeloid leukemia
Rizzello et al.^55^	2018	Pain on eye	Amphotericin B and voriconazole	*F. solani*	Acute lymphoblastic leukemia
					Neutropenia
Anandi et al.^56^	2005	Breast abscess	Ketoconazole	*F. solani*	*Diabetes mellitus*
Gardner et al.^57^	2005	Pruritic plaque on forearm	Amphotericin B and voriconazole	*F. solani*	Neutropenia
Karam et al.^58^	2005	Cutaneous nodules	Voriconazole	*F. moniliforme*	Myeloblastic leukemia

We observed that *F. solani* prevails as the etiological agent of fusariosis. The treatment also did not change over the years, indicating that amphotericin B, voriconazole, and posaconazole are prophylactic agents and treatment options for fusariosis^44^. The clinical forms of the disease in the case reports focused more on infections that present cutaneous lesions, which is characterized by the spread of the disease in patients with hematological dysfunctions.

**FIGURE 1:**
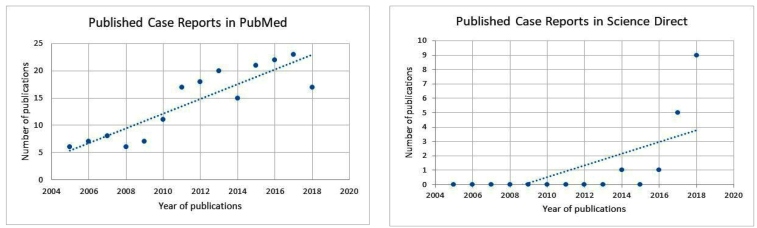
(A): Case reports of fusariosis published on the Pubmed Plataform between 2005 and 2018. (B): Case reports of fusariosis published on the Science Direct platform between 2005 and 2018

The increased incidence of fusariosis from 2005 to 2018 can be observed in Figure 1 (A-B), which graphically shows the increase in the number of articles published on the PubMed and Science Direct platforms in this time period.

## CONCLUSION

The efficient mechanisms of the dispersion of *Fusarium* spp. have led to the global distribution of clinical and environmental isolates. *F. solani* and *F. oxysporum* are the most common complexes. Infections in humans range from superficial to disseminated, and patients with hematological malignancies are the most susceptible. Dissemination of the fungus is seen mainly in immunocompromised patients because of the ease of infection related to the portal of entry of the fungus in the host, such as via the airways or the rupture of tissues and mucous membranes.

Invasive *Fusarium* infections stimulate an inadequate response by the host towards the infection, which accounts for the high mortality caused by this fungus. As such, biofilm formation renders treatment more difficult. *Fusarium* spp. exhibit intrinsic resistance to echinocandins, and some isolates exhibit resistance to azoles. In this scenario, the drugs of choice are amphotericin B and voriconazole, and drug combinations are an important means to combat multi-drug resistance. Just as the determination of the minimum inhibitory concentration provides an overview on *in vitro* resistance, it can also be considered strong evidence for selecting an antifungal treatment. Low investment by the pharmaceutical industry towards developing drugs to combat these infections was observed. 

Risk factors of individuals contribute to the occurrence of new cases and *F. solani* continues to be the main etiological agent of fusariosis. Treatment also has not changed over the years, because of the lack of research in the development of new therapeutic options for the treatment of this infection. The increased incidence of fusariosis, as reported in the articles published between 2005 to 2018, is evidence of the emergence of this fungus.
